# Development and Evaluation of Calcium-Fortified Multi-Millet Biscuits: A Nutritious Alternative to Refined Wheat Flour

**DOI:** 10.3390/foods13111696

**Published:** 2024-05-28

**Authors:** Mili Manchanda, Divya Rawat, Abhishek Chandra, Ramesh Kumar Saini

**Affiliations:** Department of Allied Health Sciences, School of Health Sciences and Technology, UPES, Dehradun 248006, Uttarakhand, India; milimanchanda.10@gmail.com (M.M.); abhishek.chandra@ddn.upes.ac.in (A.C.); rameshkumar.saini@ddn.upes.ac.in (R.K.S.)

**Keywords:** fortification, proximate analysis, sensory evaluation, response surface methodology (RSM), finger millet, pearl millet, buckwheat

## Abstract

Millet products have garnered global recognition for their exceptional nutritional profile, appealing to various age demographics, and, therefore, fortifying such products with minerals can ensure nutritional security. This research explores the feasibility of utilizing millet as a substitute for refined wheat flour in biscuit production. Three distinct millet varieties were investigated: finger, pearl, and buckwheat. Employing response surface methodology (RSM), the optimal ratio of these flours was determined, resulting in a blend of 1.5:1:1, respectively. The optimized multi-millet biscuits were further enhanced with calcium fortification and subjected to comprehensive physico-chemical analysis. Proximate composition analysis revealed favorable levels of protein (5.472 ± 0.31%), ash (2.80 ± 0.57 g/100 g), and energy density (5.8015 ± 0.004 kcal/g), indicating a significantly higher protein content, enriched mineral profile, and high energy density as compared to refined wheat flour products. Sensory evaluation encompassing attributes such as color and texture and organoleptic assessment using a nine-point hedonic scale demonstrated favorable acceptance. Additionally, the overall acceptability of the biscuits remained consistently high throughout storage, ranging from 8.263 ± 0.65 (day 0) to 8.053 ± 0.85 (day 14). This study underscores the potential of multi-millet biscuits as a nutritious and palatable alternative to traditional wheat-based snacks, offering an avenue for diversifying dietary options and promoting healthier food choices.

## 1. Introduction

Millets, small-seeded cereals, are members of the Poaceae family [1]. They are classified as major millets and minor millets. Major millets include sorghum (*sorghum bicolor*), finger millet (*Eleusine coracana*), and pearl millet (*Pennisetum glaucum*). Minor millets include proso millet (*Panicum miliaceum*), kodo millet (*Paspalum scrobiculatum*), little millet (*Panicum sumatrense*), and foxtail millet (*Setaria italica*) [2]. Minor millets have recently gained global recognition for their potential in addressing food and nutritional security issues. The United Nations proclaimed 2023 the “International Year of Millets” in recognition of their potential contribution to nutritional and health security and their resilience to climate change [3].

The production and consumption of millets are concentrated in emerging nations, particularly those in Asia and Africa. India produces the most millets, accounting for 26.6% of global production and 83% of Asian production. Millets have long been a mainstay of tribal food in the Indian states of Odisha, Madhya Pradesh, Jharkhand, Rajasthan, Karnataka, and Uttarakhand [4]. Millets have several other benefits in addition to their cultivating advantages. They are highly nutritional and rich in proteins, dietary fibers, calcium, and polyphenols, which contrast with major cereals, wheat, and rice [5]. According to epidemiological studies, millet lowers the risk of major health issues like heart disease, diabetes, and cancer. It also improves the digestive system, detoxifies the body, boosts immunity and respiratory health, gives more energy, and strengthens the muscles and nervous system. In addition to this, there is evidence that it also protects against several degenerative diseases like metabolic syndrome and Parkinson’s disease [6].

Due to these benefits, they contribute widely to various food industries, like the bakery industry, which is growing rapidly. In recent years, millet’s market value has increased due to processing. The current growth in millet’s market value can be attributed to its potential health benefits and processing, which produces various gluten-free ready-to-eat foods and nutritious supplements. Additionally, processing can aid in extending the shelf life of the millets and opening up new markets for non-traditional millet consumers [7]. Of all the bakery products, biscuits, a well-known cereal food product, are one of the most popular items as they are economically a good choice. They are extremely popular among a considerable proportion of urban and rural populations, and their demand and consumption are skyrocketing. India is the world’s second-largest biscuit manufacturer after the United States of America [8].

In this study, three millet varieties were used to develop biscuits: finger millet, pearl millet, and buckwheat. The most popular millets in India are finger millet (ragi/madua), pearl millet (bajra), and buckwheat (kuttu), which serve as a primary staple food for those living in marginal areas with few financial resources. These offer affordable food options with relatively high nutrients. The protein content of finger millet, pearl millet, and buckwheat ranges from 7.3 to 11.34 g/100 g. The fiber content of finger millet, pearl millet, and buckwheat ranges from 9.35 to 11.4 g/100 g. The calcium contents of finger millet, pearl millet, and buckwheat are 344 mg/100 g, 42 mg/100 g, and 56.6 mg/100 g, respectively [9,10,11,12,13,14,15,16].

Currently, available biscuit formulations have specific downsides because of their high fat content, low dietary fiber content, and high sugar content—all of which contribute to their high calorie content. To overcome these limitations in this study, biscuits were formulated using millet, replacing traditional ingredients like refined wheat flour with millet flour and refined sugar with jaggery powder to increase the nutritive value of biscuits. However, the final quality and acceptability of the biscuits are significantly influenced by the quantity of each ingredient in the formulation. Therefore, using a statistical model is crucial to determining the ideal amount of each ingredient that will result in a satisfactory product. The most popular method for these kinds of investigations is the response surface methodology (RSM). It is a statistical tool suitable for use in the design of experiments for food product development and optimization [17].

To determine the optimal ratio for developing biscuits, this study was designed to assess the appropriate amount of different millet flours. It also aims to understand the potential of millet as an ingredient for the development of novel food products that can be considered food for the future.

## 2. Materials and Methods

### 2.1. Materials

Finger millet, pearl millet, and buckwheat flours, butter (AMUL brand), jaggery, beetroot, and baking soda were procured from the local market of Prem Nagar, Dehradun, India. All analytical grade reagents and standards (calcium carbonate food-grade salt, potassium sulfate, copper sulfate, sulfuric acid, sodium hydroxide, boric acid, petroleum ether, ethanol, and nitric acid) were procured from HI-Media Laboratories Pvt. Ltd., Mumbai, India.

### 2.2. Preparation of Fortified Multi-Millet Biscuits

**Figure 1:**
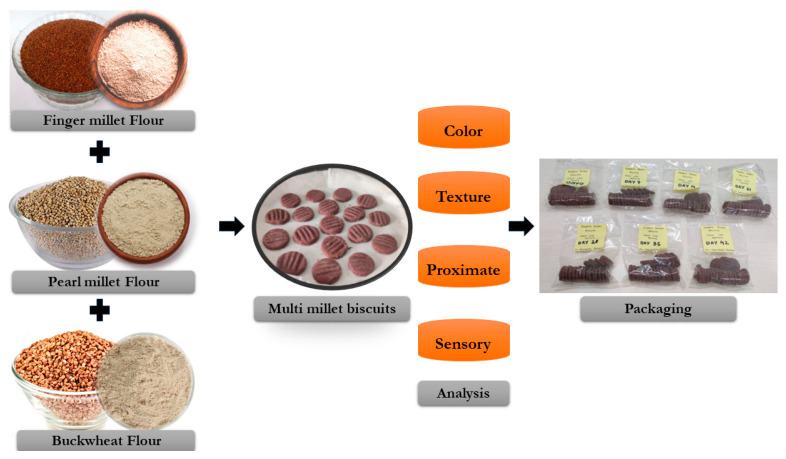
The schematic diagram for the development of multi-millet biscuits.

Biscuits were made utilizing the process recommended by Eyenga et al. [17] with minor modifications. The calcium carbonate (0.2246 g) was mixed with dry ingredients such as multi-millet flour (75 g), baking soda (0.5 g), and jaggery powder (30 g). Further, it was blended with butter (40 g) and beetroot extract (5–10 mL) in a bowl to form a dough. It was rested for 20 min at 4 °C followed by sheeting with a rolling pin with a uniform thickness range of 3–3.5 mm. The biscuits were given shape with a round-shaped metal cookie cutter of 3 cm in diameter. The molded dough was baked in an oven at 180 °C for 15–20 min. A brown bottom was an indicator of the completion of the baking process [17]. Biscuits were placed in polypropylene (PP) plastic bags measuring 51 μm, cooled at room temperature, and then heat-sealed using a sealing device (Sepak Beyond Packaging) for further testing. The schematic diagram for the development of multi-millet biscuits is provided in Figure 1.

### 2.3. Experimental Design for Optimization of Multi-Millet Flour

Central composite design (CCD) and response surface methodology were utilized to evaluate the impact of three components, ragi flour (RF); bajra flour (BF); and buckwheat flour (BWF), on six different responses, spread ratio (SR); calcium (Ca); iron (Fe); magnesium (Mg); selenium (Se); and overall acceptability (OAA). The models that best fit the experimental data were produced using Design-Expert version 13.0.9.0, and the response surface plots and analysis of variance (ANOVA) results were employed for improved accuracy. Each factor was assigned the same low level (0.5) and high level (1.5), keeping all other ingredients in the recipe constant. The 20 different experimental runs were carried out with six factorial points and six replicates of central points. The runs were carried out at random to reduce the impact of unexplained variables in the observed responses caused by outside influences [18].

### 2.4. Physico-Chemical Characteristics

#### 2.4.1. Physical Properties

##### Dough and Biscuit Yield

Following the standardized formulation of optimized multi-millet biscuits (OMBs), the dough was kneaded as per 100 g of the end product. The weight of the dough (g) was recorded using an open-top weighing balance. After sheeting and baking the dough, the weight of individual biscuits (g) was recorded using a weighing scale. A mean of 10 values was taken, and values were estimated. The percentage baking loss of OMBs was calculated by dividing the dough weight by the weight of biscuits produced with that dough after baking. An average of 10 readings was recorded.

##### Thickness, Diameter, and Spread Ratio

With the help of a scale, five biscuits were laid edge-to-edge to determine the diameter (mm) of each biscuit. The scale was then rotated 90 degrees before a new measurement was taken. Biscuit thickness was estimated by stacking five biscuits on top of one another, and height was recorded with a scale. The average diameter and thickness of the biscuits were recorded by dividing by five. Similar readings were taken for three different sets of biscuits. The SR of the OMBs was calculated with the diameter-to-thickness ratio [19].

#### 2.4.2. Functional Properties

Oil absorption capacity (OAC), bulk density (BD), water absorption capacity (WAC), and foaming capacity (FC) were calculated by the method of Sharma et al. (2016) [20]. For swelling capacity (SC) and swelling index (SI), the method of Kaushik et al. (2018) was used [21].

#### 2.4.3. Proximate Analysis

The proximate composition, including moisture, crude protein, and crude fat, was determined using AOAC methods [22]. Ash content, crude fiber, and carbohydrates were estimated by methods prescribed by Raghuramulu et al. [23]. Total energy was calculated using a digital bomb calorimeter (SPAN Automation, Model SABC-01SAS Nagar, Punjab, India) wherein 1g of powdered sample was weighed accurately and formed into a tablet. It was then placed in the calorimeter for analysis of energy value.

#### 2.4.4. Mineral Analysis

Inductively coupled plasma–optical emission spectroscopy (ICP-OES) (Analytik Jena, Thuringia, Germany, 2015) was used for metal determination of OMBs, as suggested by Hammer et al. [24] with minor modifications. Digestion was performed for both fortified and non-fortified OMB samples. The standards of concentration of 20, 10, 5, 1, and 0.1 ppm were prepared with 2% nitric acid for calibration and a blank. After calibration, the fortified and non-fortified samples of biscuits were analyzed for the following minerals: Ca, Fe, Mg, Se, K, P, Zn, Na, and Mn [24].

### 2.5. Color Analysis

Color measurement of OMBs (surface and bottom) was carried out using a colorimeter (Konica Minolta Chroma meter CR-400, Tokyo, Japan). The colorimeter was calibrated using a standard white plate. The color values L*, a*, and b* were measured using CIE coordinates using optical sensors. L* stands for lightness, with 100 being white and 0 being black; a* denotes redness when positive and green when negative; b* denotes yellowness when positive and blue when negative. ΔE, that is, the color difference, was also recorded for each sample [25].

### 2.6. Texture Profile Analysis

Textural attributes of OMBs, like hardness and fracturability, were analyzed using the TA.HD plus Universal Texture Analyzer (Stable Micro Systems, Godalming, Surrey, UK). A heavy-duty platform, a 3-point bend ring, and a 5 kg load cell were used. The pre-test, test, and post-test speeds were set to 2.0 mm/s, 1.0 mm/s, and 2.0 mm/s, respectively. The test mode was set to compression, and the trigger force applied was 5.0 g. The peak force was recorded as the hardness and fracturability of the biscuits. The textural change in OMBs was studied for the fresh batch (Day 0) and last batch (Day 21). An average of four biscuits were analyzed for each Day 0 and Day 14 sample [26].

### 2.7. Rheology

#### 2.7.1. Farinograph

With a few modest changes, a farinograph (Perten, Dough Lab 2500) was used to analyze the dough mixing characteristics of OMB flour according to AACC-approved methods [10]. On the graph, a curve was noted by the farinograph. The curve was centered on 500 farinograph units (FU) by adding water, and it ran until the curve left the 500 FU line. Water is added to the flour until it reaches a consistency of 500 Brabender units (BU), which is usually the desired consistency. A constant dough weight procedure was used in which dough weight of 480 g and 225 mL water were used to obtain absorptions on a 14% moisture basis to the nearest 0.1%. To record the values of dough development time, water absorption, degree of softening, and dough stability, the torque values were measured and plotted as a function of time [27].

#### 2.7.2. Viscosity

The rheology of OMB dough was measured with a rheometer (Anton Paar, RheolabQC, Graz, Austria) using concentric cylinder geometry. The dough sample was added up to the marked ring in the measuring cup. The cup was inserted into the measuring system (measuring bob), and the bayonet ring was turned clockwise. The temperature during the experiment was ambient. The shear rate was kept constant at 100 1/s. Rheological data were generated in terms of viscosity, shear stress, and torque with 50 recording points at regular intervals of 6 s for 5 min.

### 2.8. Packaging and Storage

For future analysis, OMBs were packaged in PP plastic pouches (51 μm). Twenty biscuits were packed in a pouch, each packet weighing approximately 100 g. The pouches were heat-sealed with a pack sealing machine and stored at ambient temperature (30 ± 4 °C). Each packet was drawn at an interval of 7 days for organoleptic evaluation.

### 2.9. Sensory Analysis

A sensory test was performed twenty-four hours after OMBs were formulated. Twenty semi-trained panelists selected for this sensory assessment study were postgraduate and research scholars from the Food and Nutrition Department at UPES University. They had sufficient knowledge regarding the descriptive sensory evaluation of biscuits. These assessors are set up, trained, and supervised according to the broad guidelines provided by ISO-norm 8586 [28]. Several basic criteria were satisfied by this panel, including availability, communication skills, food description ability, health, and motivation. They are regular in terms of the consumption frequency of biscuits and are non-allergic to any ingredients used to make biscuits. They were screened based on a triangle test performed with control samples. Hence, it was ensured that they were consistent with the skill of recognizing taste and odors related to biscuits. All of the panelists signed an informed digital consent form as volunteers. The biscuits were rated on six separate characteristics: color, taste, appearance, flavor, texture, and overall acceptability using a nine-point hedonic scale (1 = highly dislike, 5 = neither like nor dislike, and 9 = extremely like). The selected panelists were asked to evaluate the quality of biscuits at regular intervals of 7 days. Lukewarm water was provided to rinse the palate before evaluations [28].

### 2.10. Statistical Analysis

With the use of the Design-Expert^®^ 13.0.9.0 software (Stat Ease Inc., Minneapolis, MN, USA), data were analyzed using the least-squares approach, and response surfaces were produced. ANOVA was performed to assess each variable’s significance (*p* ≤ 0.05) and to confirm the model’s suitability. Every experiment was conducted in triplicate, and results were reported as mean ± SD.

## 3. Results and Discussion

### 3.1. Statistical Analysis for Optimization Using RSM

While studying the quality characteristics of biscuits or their ingredients’ interactions, the RSM has been used as a vital tool to minimize the number of trials for the optimization process. The second-order model is preferred in this study because of its flexibility and easy estimation of model coefficients. After selecting the second-order model, an analysis is performed to determine the model coefficients and their statistical significance, the mean response, and the optimum conditions that yield the best flour ratio for multi-millet biscuit development. After fitting the measured responses into the selected model, the accuracy of the response models is checked. The statistical significance of the models was determined using variance analysis. This response surface methodology is vital from an industrial point of view, where optimizing parameters like flour ratio and developing the best-quality multi-millet biscuit while saving time and cost are of utmost importance. Optimizing the biscuit development process is important to finding optimal conditions. It is, therefore, essential to identify the effects of individual factors as well as the combination and interaction of various factors [29]. Table 1 presents the experimental matrix of the CCD, including the different combinations of independent variables and the values of the responses assessed.

The ANOVA for the quadratic model for SR and Fe responses was found to be significant. Conversely, ANOVA for the linear model was significant for Ca and Mg responses. The model for OAA was not significant, indicating that no relationship was developed when there was a change in factors with respect to the organoleptic evaluation of experimental runs. This is unsurprising because every individual has a sense of perception towards a product. The minute differences in the change in factors, from a low level of 0.5 to a high level of 1.5, were not significant enough to determine a particular trend concerning this response. For Se, mean was the descriptor for the response. This is because the responses for Se for all the experimental trials were similar up to two decimal places. Hence, no significant difference was observed when there was a change in factors for this mineral. Therefore, no model was generated for Se. Out of the six responses, the predicted values were close to the actual (experimental) values in SR and Fe, indicating no significant difference. If the predicted values are reasonably close to the actual values noticed during the validation testing, the model is deemed to be appropriate [30].

### 3.2. The Effect of Independent Variables on Selected Responses

#### 3.2.1. Spread Ratio

**Figure 2:**
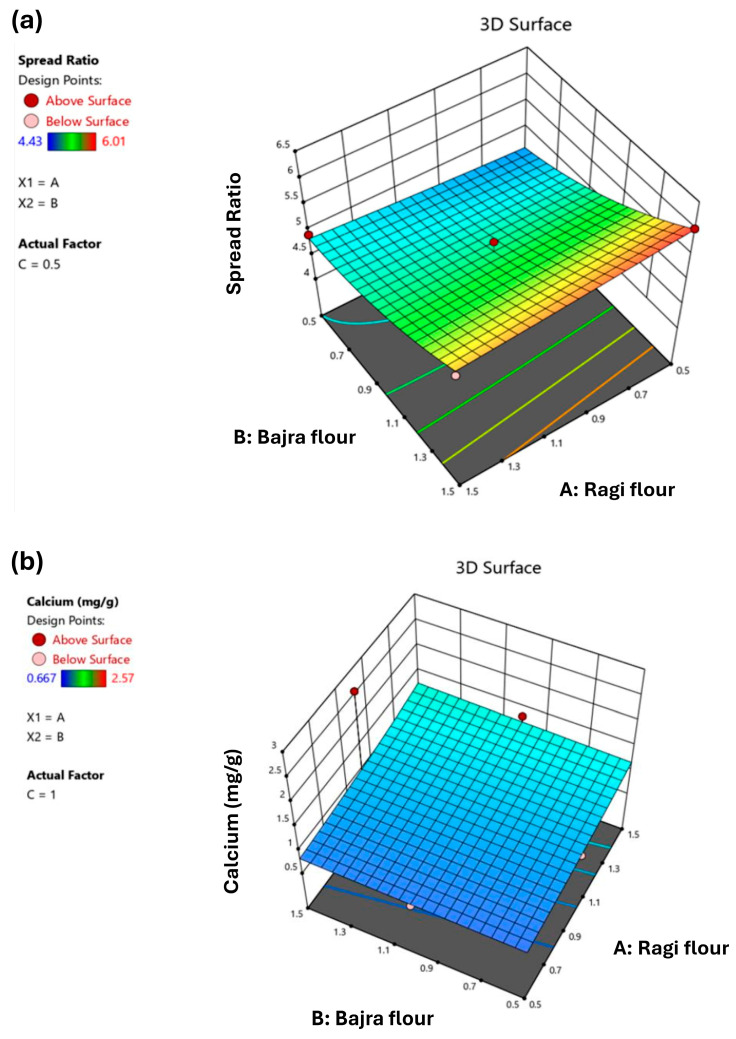
A 3D plot depicting the effect of A, B, and C on (**a**) Spread Ratio (SR); (**b**) Calcium (Ca). A = Ragi flour, B = Bajra flour, C = Buckwheat flour.

The experimental results of SR obtained are shown in Table 1. The minimum SR was measured to be 4.43 (Run 17), and the maximum was 6.01 (Run 15). The data were fitted with a second-order mathematical equation to examine the RF, BF, and BWF impact for SR. ANOVA was used to assess the model’s suitability, revealing highly significant results (*p* < 0.0015). Therefore, the second-order regression equation was thought to be capable of expressing how independent variables affected the SR of biscuits. As depicted in the 3D plot (Figure 2a), keeping BWF constant at a low level of 0.5, the trend shows an increase in SR when RF decreases and BF increases. A similar trend was reported by Nasir et al. [31].

#### 3.2.2. Overall Acceptability

For each run, the mean ± SD values were calculated for all six attributes. The experimental results of OAA are presented in Table 1. The minimum score recorded for OAA was 6.2 (Run 17), with a maximum of 7.4 (Run 8). Since the model was not significant, no relationship was built between the variables RF, BF, and BWF observed with respect to OAA.

#### 3.2.3. Minerals

**Figure 3:**
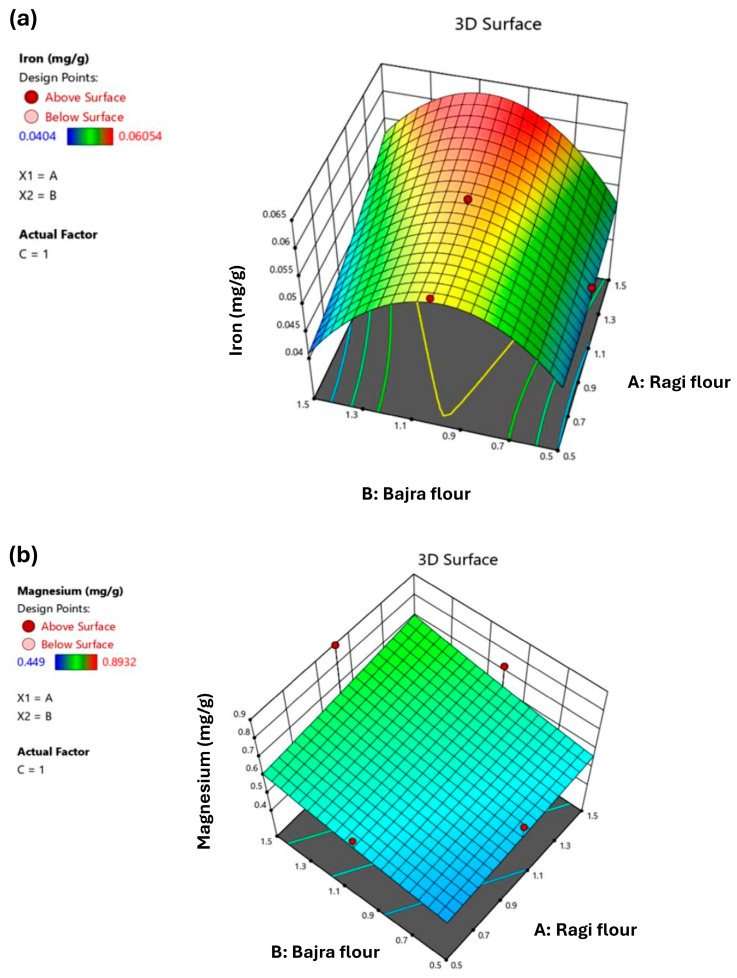
A 3D plot depicting the effect of A, B, and C on; (**a**) Iron (Fe); (**b**) Magnesium (Mg); A = Ragi flour, B = Bajra flour, C = Buckwheat flour.

The experimental results of Ca, Fe, Mg, and Se are reported in Table 1. The minimum values for Ca, Fe, Mg, and Se were 0.667, 0.0404, 0.449, and 0.02908 mg/g, respectively. The maximum values for Ca, Fe, Mg, and Se were 2.57, 0.06054, 0.8932, and 0.03163 mg/g, respectively. ANOVA evaluated the inverse transformation linear model for Ca with a significant *p*-value of 0.0379. A 3D surface plot for inverse transformation for this response is presented in Figure 2b. The data were fitted with a second-order mathematical equation to examine the impact of RF, BF, and BWF for Fe. ANOVA was used to assess the model’s suitability and was very significant (*p* < 0.0001). As a result, it was determined that the second-order regression equation could express how independent variables affected the SR of biscuits. As shown in the graph *(*Figure 3a), keeping BWF constant at a central value of 1.0, the trend observed was that, with an increase in RF and a decrease in BF, the Fe value is maximum. This is because finger millet is a richer source of Fe than pearl millet [32]. Hence, the results are desirable. ANOVA evaluated the inverse transformation linear model for Mg with a significant *p*-value of 0.0479. A 3D surface plot for inverse transformation for this response is presented in Figure 3b. The model suggested the mean for this response. Therefore, no model was generated since responses were mostly similar for all experimental runs with a standard deviation of 0.0007.

### 3.3. Regression Equations and Model Fitting

The regression coefficient (r^2^), adequate precision, and *p*-value for optimization of the multi-millet flour mixture were obtained through RSM. The models for SR and Fe were quadratic and those for Ca and Mg were linear. No model was significant for OAA and Se. The *p*-value for all responses was <0.0479, except for OAA, indicating the significance of the models. The mean and standard deviation of each response were also measured. The determination coefficient (r^2^) values for the responses were 0.8797, 0.5922, 0.4006, 0.9403, and 0.3815, respectively. The values for SR and Fe indicate reasonable precision between the data and the model. The signal-to-noise ratio for a model is measured by adequate precision. The ideal ratio, which denotes a strong signal, is greater than 4. As a result, all models can be used to explore the design space because every response has a sufficient adequate precision of larger than 4. Hence, all models are significant and valid for this experimental design. Regression coefficients and model statistics are summarized in Table 2.

### 3.4. Optimization of Multi-Millet Flour Ratio and Model Validation

The ratio of the multi-millet flour mixture used for making gluten-free biscuits was optimized manually based on the data responses and its corresponding model statistics. The optimized flour ratio was 1.5 RF, 1 BF, and 1 BWF, which was observed in Run 13. This run had the best overall responses of OAA, Ca, Fe, and Mg compared to all the experimental runs. Therefore, it can be inferred that the optimized flour ratio contributes to overall nutritional value and consumer preference. However, SR was not high for this particular run due to increased RF. A model is deemed adequate when the projected and actual (experimental) values seen during the validation tests are near each other [30]. The SR and Fe results reveal no significant difference between actual and predicted values, thereby validating the models.

### 3.5. Physico-Chemical Characteristics

#### 3.5.1. Physical Properties

The physical characteristics of OMBs are mentioned in Table 3a. A dough weight of 114.24 ± 1.98 g yields approximately 100 g of the end product. The weight of an individual biscuit was 5.27 ± 0.34 g, with a thickness of 7.966 ± 0.10 mm and a diameter of 36.36333 ± 0.24 mm.

The biscuit spread ratio depends upon sugars as they dissolve during baking, allowing an increase in diameter [19]. However, a slightly smaller diameter of these OMBs may be due to the small cookie-cutter size used while sheeting the biscuits. The thickness of baked products depends on the gluten development, which contributes to the rise in the height of biscuits [19]. The ingredients used in making these functional biscuits were gluten-free; hence, a low thickness of biscuits is desirable compared to conventional biscuits made of refined wheat flour.

Spread ratio is the ratio of diameter to thickness [19]. The higher the spread ratio, the more desirable are the biscuits. The SR of OMBs was found to be 4.568528 ± 0.032, which is similar to the results reported by Kulthe et al. [33] for cookies prepared by substituting refined flour with pearl millet in different percentages, where the spread ratio range was 3.44 to 5.05. The percentage baking loss while formulating biscuits was found to be 12.44 ± 1.52%. Similar results were reported by Dogan, 2006, where the baking loss was within the range of 10.2% to 14.5% depending on the type of biscuits formulated [34].

#### 3.5.2. Functional Properties

The functional properties of foods and flours are affected by a variety of food components, such as carbohydrates, lipids and oils, proteins, moisture, ash, fiber, and additional nutrients or diet additives added to the food. Food functionality is a property of food ingredients other than nutritional quality that significantly impacts their use and application, as well as how they affect the final product in terms of taste, appearance, and feel [35]. The OMBs were analyzed for their functional properties, as mentioned in Table 3b.

BD for OMBs was found to be 0.668 ± 0.063 g/mL. WAC of OMBs was 150.565 ± 6.13%. OAC of OMBs was observed to be 175 ± 7.07%. The SI and SC of OMBs were observed to be 0.095 ± 0.007 g/g and 10 ± 1.4 mL, respectively. The foaming capacity of OMBs came out to be 0.7 ± 0.14%. It was observed that the functional properties of the ingredients significantly influenced the OMBs.

The mass of a bulk solid, including the volume of all interparticle gaps, that fills a unit volume is known as BD [36]. BD for OMBs was found to be 0.668 ± 0.063 g/mL. Based on the study reported by Twinomuhwezi et al. [35], the BD of the flour ranged from 0.99% to 1.12%. The high BD of flour shows that it is excellent for use in food preparations because it reduces paste thickness, a crucial factor in convalescent and child feeding, or as a thickener in food products [35]. Lower BD, on the contrary, as observed in OMBs is an advantage as it can be used in the formulation of supplementary or complementary foods [37].

In water absorption capacity, the ability of flour to soak up water and expand for better food consistency is expressed. Protein is thought to play a crucial role in water absorption in viscous foods, including soups, gravies, doughs, and baked products. WAC plays a vital role in food since it affects emulsification, solubility, adhesion, dispersibility, wettability, cohesion, viscosity, and gelation [35]. WAC of OMBs was 150.565 ± 6.13%. The WAC as mentioned in the literature for finger millet flour was 0.93 ± 0.06 to 1.23 ± 0.06 mL/g [38]; that of raw pearl millet flour was 226 ± 0.03% [39]; and that of buckwheat flour was 133.67 ± 0.67% [14], which are similar to that of the combined composite flour used for making OMBs. WAC is an important indicator in determining if the baked product can be used in aqueous formulations [38].

Fat binding by the non-polar side chain of proteins is known as oil absorption capacity. It is mostly due to the physical trapping of oils. It measures how quickly proteins bind to fat in food formulations [35]. OAC of OMBs was observed to be 175 ± 7.07%. The high fat level in the biscuit recipe may cause this high OAC percentage. As reported in the literature, OAC for finger millet flour was 1.63 ± 0.06 mL/g [40]; that of pearl millet flour was 150 ± 0.06% [39]; and that of buckwheat flour was 181.37 ± 0.58% [14], which is similar to that of the combined composite flour used for making OMBs. The ability of food products to absorb oil is critical for nutrient and energy density, particularly in infants and young children. Furthermore, the flour’s high oil absorption capability makes it ideal for improving mouthfeel and flavor when employed in meals [35].

The swelling index is considered a quality criterion for bakery product formulation [41]. The volume in milliliters taken up by the expansion of 1 g of food material under specified conditions is known as the swelling capacity [42]. The SI and SC of OMBs were observed to be 0.095 ± 0.007 g/g and 10 ± 1.4 mL, respectively. Similar findings were observed by Adegunwa et al. [43] for 100% millet flour.

The foaming capacity of OMBs came out to be 0.7 ± 0.14%. FC refers to a substance’s capacity to generate foam following vigorous shaking in a solution [41]. Proteins are surface-active, which causes them to foam upon whipping [44]. OMBs have 5.472 ± 0.31% protein, which might not be sufficient to produce foam, hence explaining the low FC of OMBs.

#### 3.5.3. Proximate Analysis

The proximate composition of OMBs prepared from finger millet, pearl millet, and buckwheat is given in Table 3c.

Estimating moisture content and total solids is critical for food stability and quality and for predicting food behavior throughout preparation, storage, and consumption [35]. Moisture content in OMBs was 3.1 ± 0.14%. After baking, the constituents lose moisture, resulting in a decrease in the moisture content of the final product. Similar findings were observed by Salunke et al. [45] for the moisture content of biscuits made of barnyard millets. As a result, it can be kept at room temperature because of its low moisture content, which makes it less prone to microbial and fungal infections. Proteins play a role in the nutritional value, texture, and sensory aspects of foods, as well as having structural and biological roles [35]. Protein in optimized biscuits was observed to be 5.472 ± 0.31%, which is similar to that estimated using Indian Food Composition Tables (IFCTs) for the biscuit sample. Eneche [46] reported similar findings for biscuits made from millet flour.

A group of substances known as lipids are very weakly soluble in water but more soluble in ether, chloroform, and other organic solvents [41]. Due to the incorporation of butter in the formulation, the fat content of OMBs was observed to be 29.5 ± 0.85 g/100 g, which is similar to that estimated by IFCTs. Fat imparts tenderness and assists in texture improvement to the product, making it more palatable and acceptable to consumers. Florence et al. [47] reported similar results.

The ash content of food indicates the total amount of mineral elements present in the food [35]. Ash of OMBs was observed to be 2.8 ± 0.57 g/100 g. The increase in ash from individual raw materials, 1.217 g/100 g, as calculated with IFCTs, to the baked product might be due to the use of a leavening agent, baking soda, in the formulation or baking at a high temperature. Eneche [46] recorded similar findings for biscuits composed of millet flour. High ash content in biscuits could imply a high quantity of minerals in the product.

Crude fiber, which refers to the residue after acid–alkali digestion, was found to be 0.741 ± 0.008 g/100 g for OMBs. Omah [48] mentioned similar results for biscuits made of pearl millet flour. It aids in the prevention of heart disease, colon cancer, diabetes, and other ailments. Crude fiber reduces the rate of blood glucose absorption and intercolonic pressure, which reduces the risk of colon cancer [41].

Carbohydrates calculated by the difference method were 58.387 ± 0.17 g/100 g, which is close to the estimated value of 53.472 g/100 g for raw materials using IFCTs. The high carbohydrate content of biscuits suggests that they could effectively address protein–energy malnutrition (PEM), as carbohydrates supply energy to the body while allowing protein to be spared. Instead of being used as a source of energy, protein can be employed for its basic activities of creating and mending worn-out tissues. Breakfast meals and weaning formulas should have a high carbohydrate content. Hence, these biscuits could be used for such meals [41].

The energy value of OMBs was found to be 5.8015 ± 0.004 kcal/g. The high-calorie content might be due to the formulation’s incorporation of butter and jaggery. Readings were close to the one calculated using IFCTs. The analysis of different parameters in this study demonstrates that the developed multi-millet biscuits are rich in fiber and minerals, making them a healthier alternative to the existing products made from refined wheat flour. They also have the potential to be incorporated into mid-day meal schemes initiated by the Government of India.

#### 3.5.4. Mineral Analysis

The optimized biscuit samples were evaluated for minerals (Ca, Fe, Mg, Zn, and Na) using ICP-OES. The results are shown in Table 3d. Ca for the fortified sample was 1.462 ± 0.051 mg/g, and that of the non-fortified sample was 1.256 ± 0.015 mg/g. A significant difference of 0.206 mg/g was observed between the Ca content of the fortified and non-fortified samples. This indicated the effect of fortification with calcium carbonate in the formulation. The observed values for biscuit samples were 0.16 ± 0.007 mg/g, 0.682 ± 0.005 mg/g, 0.15 ± 0.002 mg/g, and 1.49 ± 0.03 mg/g for Fe, Mg, Zn, and Na, respectively. As reported in the literature, finger millet is the richest source of minerals among cereals and millet. Also, buckwheat grains are a good source of microminerals. Ca (110 mg/100 g), Mg (390 mg/100 g), Fe (4 mg/100 g), and Zn (0.87 mg/100 g) are all abundant in its grain [49]. This explains the high levels of micronutrients in the biscuits.

### 3.6. Color analysis

Color is a chief attribute for the selection and acceptability of bakery products [50]. OMBs incorporated with beetroot extract were evaluated for color attributes, from both the surface and bottom, in terms of L*, a*, b*, and ΔE. The surface’s L*, a*, and b* values were 39.12 ± 0.16, 11.42 ± 0.14, and 10.12 ± 0.14, respectively. The same values for the bottom of the biscuit were observed to be 41.75 ± 0.14, 10.56 ± 0.25, and 14.36 ± 0.04, respectively. The trend shows an increase in lightness (L*), a decrease in redness (a*), and an increase in yellowness (b*) from the surface to the bottom of the biscuit. A high L* value for the bottom indicates a lighter color than the surface. This is unsurprising because the surface was darker due to the beetroot color, whereas the bottom was brown after baking. A positive and higher a* value for the surface indicates more redness when compared to the bottom. This is true because beetroot is redder in color, which is a major contributor to the color of the biscuit surface. Conversely, a positive and higher b* value for the bottom indicates more yellowness at the bottom when compared to the surface. The total color difference (ΔE) of the surface (20.16 ± 0.14) and bottom (15.74 ± 14) of OMBs from the standard was high, confirming that it had a distinct color. ΔE values > 12.0 indicate a very obvious color difference [51].

### 3.7. Texture Profile Analysis

The texture is a primary characteristic of bakery products and contributes to evaluating the product’s overall acceptability [33]. The bending or snap test was used to analyze the texture of OMBs. Hardness and fracturability were used to represent firmness. The distance at break (of the biscuit) and the maximum peak force detected in the curve served as indices of hardness (g) and fracturability (mm), respectively. Once the trigger force was reached, the force continued to rise until it reached its maximum peak, at which point the biscuit broke into two pieces. This maximum force was expressed as the “hardness” of the biscuit sample. The distance at the point of the break represented the biscuit’s bending resistance. This represented the “fracturability” of the biscuits [52]. In the snap test, the hardness value for freshly baked, Day 0, biscuits was found to be 2318.45 ± 852.2 g, which decreased significantly on Day 14, the value being 904.66 ± 210.54 g. Likewise, the Day 0 sample was broken at a higher distance of 7.93 ± 0.82 mm and had a low fracturability, whereas Day 14 stored biscuits were broken at a shorter distance of 4.28 ± 4.34 mm, indicating high fracturability. Comparable values were reported by Saklani et al. and Patil et al. [26,52]. This indicates that upon storage, the texture of OMBs in terms of hardness and fracturability decreased significantly. Kulthe et al. [33] also conducted studies on the textural properties of cookies made of pearl millet flour.

### 3.8. Rheology

#### 3.8.1. Farinograph

A farinograph is used to analyze the dough-making properties of the flour used in formulating bakery products [10]. In the farinograph equipment (Perten, Dough Lab 2500), the mixer was subjected to defined mechanical stress by mixer blades. The resistance of the dough developed against the blades depends on the dough viscosity, measured as torque. It was represented as a function of time in a diagram that records water absorption values, dough development time (DDT), dough stability, and degree of softening [53]. The multi-millet flour mixture, optimized using RSM and used in the formulation of OMBs, was analyzed by the farinograph. The peak reached 339.6 FU, with water absorption and dough development time of 52.8% and 9.9 min, respectively. As reported by Thorat and Ramachandran [53], with the addition of millet flour, the water absorption decreases due to the dilution or lack of gluten. In addition to this, with an increase in millet flour, the dough development time also increases. Since the flour used for OMBs was gluten-free, lower water absorption and longer dough development time are desirable. Dough stability is an index of dough strength, which tends to decrease with an increase in millet flour [53]. The multi-millet flour mixture used for farinograph analysis reported dough stability as 3.4 min. Similar results were reported by Saha [10]. Lastly, the degree of softening denotes the elastic proportion of the dough, which tends to decrease with an increase in millet flour [53]. This explains the lack of results for the degree of softening of flour used for OMBs; since it is made entirely of millet, it lacks elasticity.

#### 3.8.2. Viscosity

The rheological properties were measured in terms of viscosity (Pa·s), shear stress (Pa), and torque (mNm). It was observed that the viscosity decreased with an increase in time, indicating that biscuit dough was a non-Newtonian fluid. In addition to this, the shear stress and torque also decrease with increasing time. Such a trend is observed in thixotropic fluids. Thixotropy occurs when a material is subjected to a higher shear rate, which is a reversible, isothermal, time-dependent decrease in apparent viscosity [54].

### 3.9. Sensory Analysis

**Figure 4:**
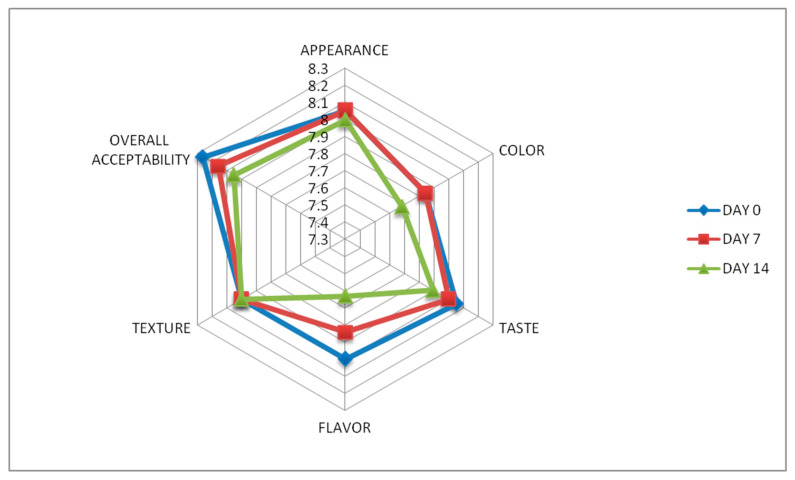
Radar chart for sensory analysis of OMBs over the period of 14 days at regular intervals.

An important factor in predicting consumer reaction and product acceptability is the organoleptic evaluation of a food product. OMBs were evaluated using a nine-point hedonic scale on six different attributes: appearance, color, taste, flavor, texture, and overall acceptability, at an interval of 7 days for 2 weeks. The results are recorded in Table 4. Appearance and color did not show any difference from Day 0 to Day 7 of the evaluation. The mean scores were calculated to be 8.053 ± 0.85 and 7.842 ± 1.07, respectively. However, a slight decrease in these two parameters was observed for Day 14 OMB samples, the mean score being 8 ± 0.94 and 7.684 ± 0.95, respectively. Taste and flavor decreased moderately over time. The scores on Day 0, Day 7, and Day 14 were observed as 8.053 ± 0.91, 8 ± 1.05; 8 ± 0.82, 7.842 ± 1.01; and 7.895 ± 1.05, 7.631 ± 1.12, respectively. The texture of OMBs did not change over the storage period, the mean value being constant at each interval of evaluation, that is, 8 ± 0.82. Lastly, the overall acceptability decreased moderately at each interval, starting from 8.263 ± 0.65 to 8.158 ± 0.69 and finally to 8.053 ± 0.85. The trend is shown in Figure 4. It indicates that storage did not affect any major or significant changes in evaluating the organoleptic properties of OMBs over the period of two weeks [55]. These biscuits’ crisp and crunchy texture added to their organoleptic appeal and made them even more delectable.

The discussed results showed that in addition to texture, one of the most important factors influencing consumer preference and acceptability of biscuits is the perception of freshness. Analyzing the rheological properties of the dough is crucial because a stickier dough probably impacts the consumer’s choice for the texture attribute. Sensory analysis is pivotal to assessing product quality and consumer acceptability. Color, visual appearance, flavor, taste, and texture have the most significant initial impact on consumers. The results showed that the color, texture profile, dough rheology, and sensory evaluation are important for the consumers’ overall acceptability of the multi-millet biscuits, and the findings of this study support this. The protein (5.472 ± 0.31%), ash (2.80 ± 0.57 g/100 g), and energy density (5.8015 ± 0.004 kcal/g) were all found to be at favorable levels in the composition study, showing significantly greater protein content, richer mineral profile, and high energy density. Throughout storage, the biscuits’ overall acceptability stayed high, ranging from 8.263 ± 0.65 on Day 0 to 8.053 ± 0.85 on Day 14.

## 4. Conclusions

Calcium-fortification-produced multi-millet biscuits are of higher quality in terms of fiber and minerals, particularly calcium, texture, and organoleptic acceptability. These biscuits can potentially improve many dietary deficiencies and diversify the bakery market. Moreover, due to its unique characteristic of growing with less water, millet has become more important for maintaining food and nutritional security as well as sustainability. The utilization of these indigenous millets to prepare biscuits suggests that they can be successfully incorporated into conventional biscuit recipes, replacing refined wheat flour. In this research, the indigenous ingredients shortlisted for making biscuits were optimized through RSM. The optimized finger millet, pearl millet, and buckwheat flour ratio was 1.5:1:1. This best combination was used to produce multi-millet biscuits and further assessed for the proximate, textural property, functional property, and shelf-life studies. These energy-dense biscuits are ideal for children with malnutrition and other micronutrient deficiencies.

Further studies are recommended for developing other fortified and convenient ready-to-eat snacks with better sensory attributes and shelf-life, leading to better consumer acceptance. These premixes could enhance the use of millets as a novel flour in food product development. They could contribute to uplifting micronutrient profiles in the diet. In addition, some low-cost nutritive alternatives for shortening agents could be explored to replace butter in baking biscuits. Simultaneously, this study opens the door for assessing the presence of anti-nutritional compounds and associated flavors in millets, which are the major constraints limiting their widespread consumer acceptance.

## Figures and Tables

**Table 1:** Experimental results for run conditions are defined in the central composite design (CCD) matrix for developing multi-millet biscuits.

	F1	F2	F3	R1	R2	R3	R4	R5	R6
Run	A	B	C	SR	OAA (0–9)	Ca (mg/g)	Fe (mg/g)	Mg (mg/g)	Se (mg/g)
1	1.5	1.5	0.5	5.63	7.22	0.9505	0.05513	0.6388	0.02908
2	1	1	1	4.46	6.5	0.861	0.05812	0.5514	0.03066
3	0.5	1	1	5.02	6.97	0.7716	0.05611	0.5941	0.02934
4	1.5	0.5	0.5	4.92	6.86	1.205	0.05106	0.5128	0.03066
5	1	1	1	4.48	6.5	0.865	0.05814	0.5515	0.03067
6	1	0.5	1	4.78	7.33	0.922	0.04624	0.5925	0.03015
7	1	1	1	4.49	6.4	0.865	0.05814	0.5514	0.03066
8	0.5	1.5	1.5	4.69	7.4	0.667	0.04165	0.5634	0.03158
9	1.5	1.5	1.5	4.46	6.9	1.081	0.05818	0.6644	0.03163
10	1	1	1	4.48	6.5	0.865	0.05816	0.5514	0.03066
11	1	1	1.5	4.67	6.9	1.064	0.05597	0.6696	0.0294
12	0.5	0.5	1.5	4.82	7.21	0.697	0.0404	0.449	0.03102
13	1.5	1	1	4.58	7.34	1.332	0.05932	0.7299	0.03065
14	1	1	0.5	5.27	7.04	1.008	0.06054	0.6621	0.03144
15	0.5	1.5	0.5	6.01	7.15	0.819	0.04592	0.5225	0.03087
16	1	1.5	1	5.19	7.32	2.57	0.04058	0.8932	0.03104
17	0.5	0.5	0.5	4.43	6.2	0.93	0.0512	0.551	0.03061
18	1	1	1	4.57	6.5	0.865	0.05814	0.5513	0.03066
19	1.5	0.5	1.5	4.74	7.2	1.045	0.04764	0.5077	0.03102
20	1	1	1	4.47	6.5	0.864	0.05816	0.5514	0.03066

F1, F2, F3 = Factor 1, Factor 2, Factor 3; R1, R2, R3, R4, R5, R6 = Response 1, Response 2, Response 3, Response 4, Response 5, Response 6; A = Ragi Flour; B = Bajra Flour; C = Buckwheat Flour; SR = Spread Ratio, OAA = Overall Acceptability, Ca = Calcium, Fe = Iron, Mg = Magnesium, Se = Selenium; Run 13 (highlighted) was considered best and optimized for further testing.

**Table 2:** Regression coefficient and model statistics for individual responses.

Term	SR	OAA	Ca	Fe	Mg	Se
F value	8.13	1.61	3.56	17.49	3.29	Nil
P > F	0.0015	0.2332	0.0379	<0.0001	0.0479	Nil
Mean	4.81	6.9	1.01	0.0529	0.593	0.0306
SD	0.4292	0.3785	0.3999	0.0068	0.0971	0.0007
r^2^	0.8797	0.5922	0.4006	0.9403	0.3815	Nil
Adequate Precision	11.2618	4.4401	6.3215	13.371	6.5189	Nil

Values are cumulative of 20 experimental runs. SD = Standard Deviation; r^2^ = Coefficient of Determination; SR = Spread Ratio; OAA = Overall Acceptability; Ca = Calcium; Fe = Iron; Mg = Magnesium; Se = Selenium.

**Table 3:** Physico-chemical characteristics of OMBs.

(a) Physical properties		(b) Functional properties	
Parameter	Result	Parameter	Result
Dough Weight (g)	114.24 ± 1.98	BD (g/mL)	0.66 ± 0.063
Biscuit Weight (g)	5.27 ± 0.34	WAC (%)	150.56 ± 6.13
Thickness (mm)	7.966 ± 0.10	OAC (%)	175 ± 7.07
Diameter (mm)	36.36 ± 0.24	SI (g/g)	0.09 ± 0.007
Spread Ratio	4.56 ± 0.032	SC (mL)	10 ± 1.4
% Baking Loss	12.44 ± 1.52	FC (%)	0.7 ± 0.14
(c) Proximate analysis		(d) Mineral analysis	
Parameter	Result	Parameter	Result
Moisture (%)	3.1 ± 0.14	Ca (fortified)	1.462 ± 0.051
Protein (%)	5.472 ± 0.31	Ca	1.256 ± 0.015
Fat (g/100 g)	29.5 ± 0.85	Fe	0.16 ± 0.007
Ash (g/100 g)	2.8 ± 0.57	Mg	0.682 ± 0.005
Crude Fiber (g/100 g)	0.741 ± 0.008	Zn	0.15 ± 0.002
Carbohydrate (g/100 g)	58.387 ± 0.17	Na	1.49 ± 0.03
Energy (kcal/g)	5.8015 ± 0.004		

The values are expressed as mean ± SD of triplicate determination on dry wet basis. BD = Bulk Density, WAC = Water Absorption Capacity, SI = Swelling Index, OAC = Oil Absorption Capacity, SC = Swelling Capacity, FC = Foaming Capacity, Ca = Calcium, Fe = Iron, Mg = Magnesium, Zn = Zinc, Na = Sodium.

**Table 4:** Sensory scores of OMBs for 14 days at regular intervals of 7 days.

Day	Appearance	Color	Taste	Flavor	Texture	OAA
Day 0	8.053 ± 0.85	7.842 ± 1.07	8.053 ± 0.91	8 ± 1.05	8 ± 0.82	8.263 ± 0.65
Day 7	8.053 ± 0.91	7.842 ± 1.07	8 ± 0.82	7.842 ± 1.01	8 ± 0.82	8.158 ± 0.69
Day 14	8 ± 0.94	7.684 ± 0.95	7.895 ± 1.05	7.631 ± 1.12	8 ± 0.94	8.053 ± 0.85

The values are expressed as mean ± SD of 20 semi-trained panelists.

## Data Availability

The original contributions presented in the study are included in the article, further inquiries can be directed to the corresponding author.
